# Time-varying predatory behavior is primary predictor of fine-scale movement of wildland-urban cougars

**DOI:** 10.1186/s40462-018-0140-6

**Published:** 2018-11-02

**Authors:** Frances E. Buderman, Mevin B Hooten, Mathew W Alldredge, Ephraim M Hanks, Jacob S Ivan

**Affiliations:** 1 Colorado State University, Departments of Fish, Wildlife, and Conservation Biology, 1484 Campus Delivery, Fort Collins, CO 80523 USA; 2 U.S. Geological Survey, Colorado Cooperative Fish and Wildlife Research Unit, Departments of Fish, Wildlife, and Conservation Biology and Statistics, Colorado State University, 1484 Campus Delivery, Fort Collins, CO 80523 USA; 3 Colorado Parks and Wildlife, 317 W Prospect Road, Fort Collins, CO 80526 USA; 4 Pennsylvania State University, W-250 Millennium Science Complex, University Park, State College, PA 16802 USA

**Keywords:** Animal movement, Hierarchical model, Individual variation, Population-level, Predation, Telemetry, Wildland-urban interface

## Abstract

**Background:**

While many species have suffered from the detrimental impacts of increasing human population growth, some species, such as cougars (*Puma concolor*), have been observed using human-modified landscapes. However, human-modified habitat can be a source of both increased risk and increased food availability, particularly for large carnivores. Assessing preferential use of the landscape is important for managing wildlife and can be particularly useful in transitional habitats, such as at the wildland-urban interface. Preferential use is often evaluated using resource selection functions (RSFs), which are focused on quantifying habitat preference using either a temporally static framework or researcher-defined temporal delineations. Many applications of RSFs do not incorporate time-varying landscape availability or temporally-varying behavior, which may mask conflict and avoidance behavior.

**Methods:**

Contemporary approaches to incorporate landscape availability into the assessment of habitat selection include spatio-temporal point process models, step selection functions, and continuous-time Markov chain (CTMC) models; in contrast with the other methods, the CTMC model allows for explicit inference on animal movement in continuous-time. We used a hierarchical version of the CTMC framework to model speed and directionality of fine-scale movement by a population of cougars inhabiting the Front Range of Colorado, U.S.A., an area exhibiting rapid population growth and increased recreational use, as a function of individual variation and time-varying responses to landscape covariates.

**Results:**

We found evidence for individual- and daily temporal-variability in cougar response to landscape characteristics. Distance to nearest kill site emerged as the most important driver of movement at a population-level. We also detected seasonal differences in average response to elevation, heat loading, and distance to roads. Motility was also a function of amount of development, with cougars moving faster in developed areas than in undeveloped areas.

**Conclusions:**

The time-varying framework allowed us to detect temporal variability that would be masked in a generalized linear model, and improved the within-sample predictive ability of the model. The high degree of individual variation suggests that, if agencies want to minimize human-wildlife conflict management options should be varied and flexible. However, due to the effect of recursive behavior on cougar movement, likely related to the location and timing of potential kill-sites, kill-site identification tools may be useful for identifying areas of potential conflict.

**Electronic supplementary material:**

The online version of this article (10.1186/s40462-018-0140-6) contains supplementary material, which is available to authorized users.

## Background

Individual-level movement decisions are one of the underlying processes that give rise to population-level patterns such as species distributions or their density and abundance on the landscape [1]. Movement decisions are a function of a number of variables, including the current location of the individual and the alternative available landscape [1]. Therefore, a central theme of animal ecology is the assessment of an individual’s selection for habitat, given what is available [2]. Habitat selection is typically characterized using resource selection functions (RSF), which are often fit using logistic regression to compare the locations used by an individual or population to a random sample taken across some area defined as “available” [3]. Use that is disproportionate to habitat availability implies that the individual selects for, or avoids, the given habitat [3]. However, inference on selection depends on what components are considered available to the animal [2]. For example, an animal may use a resource disproportionately less than is available in its home range, however it may have chosen its home range because the resource was abundant [2].

In addition, availability is constrained by an individual’s range of movement. To account for dynamic availability, spatio-temporal point process models simultaneously estimate the resource selection function and time-varying availability kernels, which is the area an individual is capable of moving to over a given period of time [4–6]. The more commonly used method, a step selection function, approximates the availability kernel by using conditional logistic regression and a sample of “available” steps that an individual could have taken (e.g., [7–9]). Recent methods have used conditional logistic regression to separately approximate the movement and time-varying availability kernels, in the vein of spatio-temporal point process models (e.g., [10]). However, because all of these methods are formulated in discrete time, inference is made only when data were observed and not on the unobserved path. In addition, aside from the spatio-temporal point process of [6], none of these methods account for measurement error in the observed locations.

In contrast to many resource selection studies, one of the primary goals of continuous-time movement models is to estimate the true path of an individual when it was unobserved [6, 11–14]. Continuous-time movement models can also incorporate measurement error and irregular observations in time. However, movement models are typically time consuming and computationally intensive to fit, making it difficult to obtain inference on multiple individuals [15]. If inference on multiple individuals is attainable, it may be possible to identify a population-level response that is consistent across individuals, which would provide a rigorous link between individual choices and population-level patterns [1]. In addition, understanding individual variability may help identify individuals that associate more strongly with certain features of the landscape [16].

A recently developed method, continuous-time Markov chain (CTMC) modeling, incorporates an explicit movement model to obtain information on travel speeds and directionality. Travel speeds may provide indirect inference on resource selection [17] and avoid absolute statements about selection [2]. The CTMC method [18, 19] is fit in two stages, where the first stage uses a continuous-time movement model to obtain inference on where the individual was when it was unobserved and account for measurement error, while the second stage allows for evaluation of landscape drivers of animal movement. The second stage of the analysis uses a Poisson specification with an offset to model transition rates; therefore, statistical software based on a Poisson likelihood can implement the CTMC movement model [19]. The flexibility of the CTMC framework can account for time-varying responses to landscape drivers by allowing coefficients to vary temporally [19], and it can also be implemented in a Bayesian hierarchical framework, allowing for inference on individual- and population-level drivers. Previous applications of the CTMC framework focused on inference for single individuals and did not make inference across multiple individuals [19, 20].

Quantifying individual variability in habitat selection, while simultaneously estimating population-level patterns, can be important for management and conservation issues where resources are heterogeneous or cause points of conflict [21]. Some large carnivores, such as cougars (*Puma concolor*), have undergone recent range expansions into human-modified landscapes [22], but they rarely use the heavily modified landscapes in urban and suburban areas, instead relying on the rural and exurban areas at the wildland-urban interface [21, 23]. Along with increased risk from human interactions [23], human-modified landscapes may contain greater numbers of both primary (ungulates, e.g., [24]) and secondary (domestic animals, e.g., [25]) prey for large carnivores compared to adjacent wild-land areas.

As early as 1998, the frequency of human-cougar interactions along portions of the Front Range, a mountain range extending north-south from Casper, Wyoming to Pueblo, Colorado, have increased due to encroaching residential development, increasing cougar populations, and increasing prey densities near human populations [26]. The Front Range Urban Corridor runs along the eastern edge of the Front Range, while the Front Range itself contains a matrix of towns and areas that are managed for recreational use by county, state, and federal agencies. Human-cougar interactions have remained high in recent years (Mat Alldredge, Colorado Parks and Wildlife, personal communication), and cougars have been observed using developed areas in the Front Range as a hunting ground [27, 28]. In addition, due to their desirable qualities, regions adjacent to protected areas have higher human population growth compared to growth in rural, non-protected areas [29], increasing the potential for human-wildlife conflict [30].

Given the increasing potential for human-wildlife conflict as development permeates rural and wildland areas along the Front Range and elsewhere in the West, we sought to extend previous work by explicitly modeling fine-scale cougar movement to identify key drivers of their behavior, and in doing so, better understand their use of the wildland-urban landscape in both space and time. Many cougar studies do not explicitly model movement, and instead focus on resource selection; the animal locations used for inference were sometimes obtained only during daylight (e.g., [31–33]), obtained during night and day but were treated equivalently (e.g., [34]), or obtained at unspecified times (e.g., [35, 36]). Inference on time-varying behavior has been limited to separate analyses on discretized temporal periods (e.g., [17, 22]). Some studies have also focused exclusively on kill site and hunting locations (e.g., [27]) or non-kill site locations (e.g., [17, 22]). We used the CTMC framework to model individual- and population-level cougar responses to landscape features in continuous time, which allowed for direct inference on how behavior varied at a temporally fine scale, given what was available. In addition, by using a hierarchical modeling framework, we accounted for individual-level variation, which may be a function of the spatial distribution of prey items or the behavioral flexibility of a generalist predator [21, 37], while still obtaining population-level inference across a suite of individuals.

## Methods

### Data collection and study area

**Fig. 1:**
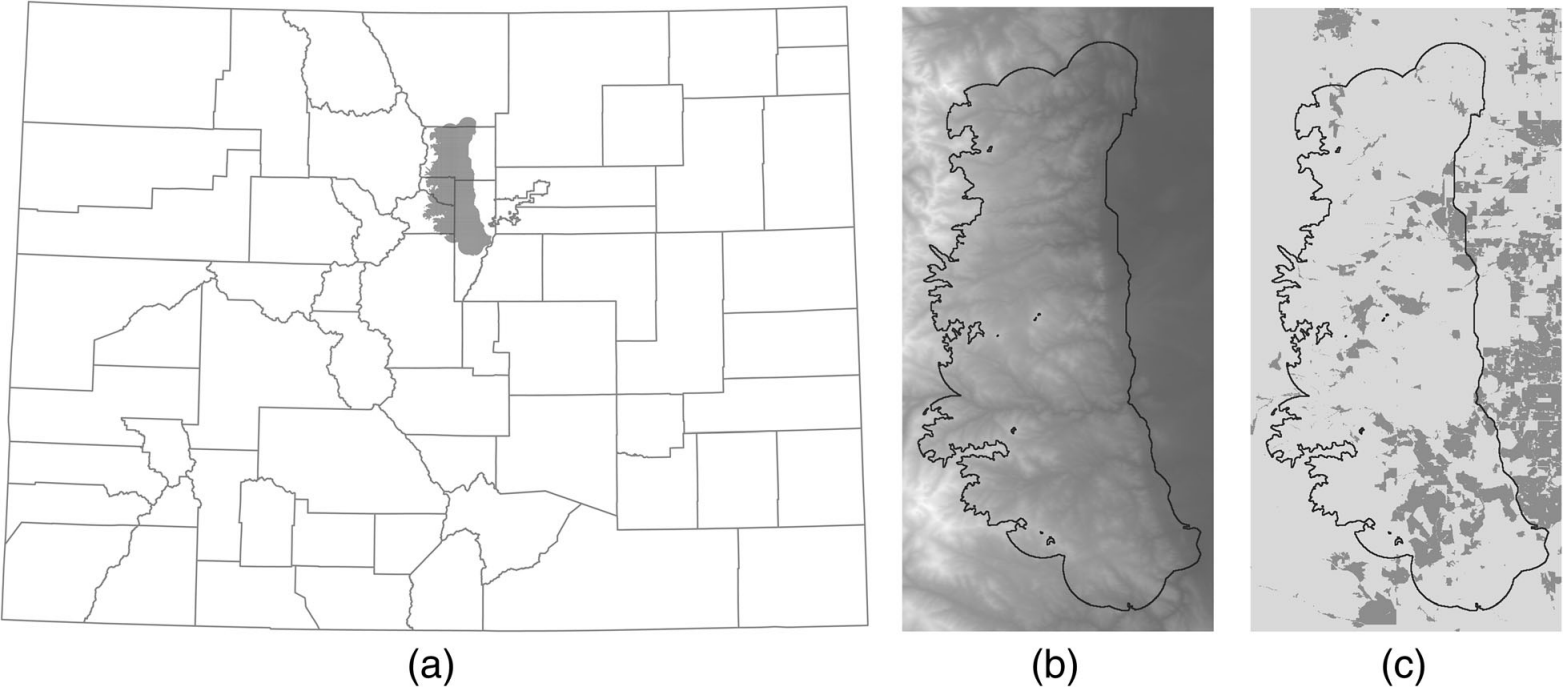
Map of Colorado counties, with the cougar movement study area plotted in gray (Fig. 1**a**). Elevation (m; Fig. 1**b**) and land classified as developed (dark gray is < 10 acres/unit; Fig. 1**c**) is shown for the study area and surrounding area

As part of an ongoing study by Colorado Parks and Wildlife (CPW), cougars were trapped and fit with global positioning system (GPS) collars and released along the Front Range of Colorado (CPW ACUC 01–2008; Fig. 1). We focused on 19 adult individuals (M = 5, F = 14) that were monitored April 1–15 2011, 21 adult individuals (M = 7, F = 14) that were monitored during June 16–30 2011, and 21 adult individuals (M = 3, F = 18) that were monitored October 1–15 2011. The time periods of interest were chosen for two reasons: first, because observations were available for a large number of individuals, which is critical for making population-level inference across individuals, and second, because we were interested in examining seasonal differences in cougar movement due to temporal variability in the landscape-level covariates. For example, we expected a strong response to prey-based covariates year round but with seasonal shifts to reflect seasonal changes in prey availability. In June, mule deer fawns are born, and form a primary prey source for cougars [38] and are at a disproportionately high risk for predation [39]. However, cougars have been observed relying on smaller prey items in April, potentially due to competition with other species, and by October fawn predation has decreased and their diet switches to deer and elk (Mat Alldredge, CPW, personal observation).

Cougars were trapped using cage traps, hounds, and foothold snares, and the minimum difference between trapping and the analysis window was 12 days, with the average time from trapping to analysis being 114 days for April, 130 days for June, and 235 days for October. One individual (AF69) was darted and relocated mid-analysis (April), however we generated the CTMC data separately for each period before making inference on movement drivers across the observation period. All individuals were monitored with Vectronics collars (Vectronics GmbH, Berlin, Germany) programmed to obtain fixes every 3 hours.

Our study area comprised a 2,700 km^2^ region in the Colorado Front Range to the north-west of Denver (Fig. 1). The study area consisted of a matrix of private (43%) and public (57%) land [27]. Private land included areas of rural, exurban, suburban development, and small towns. Public land was managed by federal, state, and municipal governments for recreational activities or as open space. Road density was, on average 1.6 km/km^2^, but ranged from no roads to 16.6 km/km^2^. Elevation ranged between 1,522 and 4,328 m, generally increasing east to west, with development decreasing along a similar gradient. Ponderosa pine, Douglas fir (*Pseudotsuga menziesii*), lodgepole pine (*Pinus contorta*), and spruce-fir (*Picea engelmannii*-*Abies lasiocarpa*) were the predominant non-agriculture vegetation types, in order of dominance from east to west. Cougar density was approximately 2.4 individuals per 100 km^2^ (Mat Alldredge, CPW, personal communication). Native herbivores (elk and deer) form the predominant component of cougar diet in the region, with a smaller contribution from domestic species (pets and livestock) and synanthrophic wildlife [40]. However, the proportion of domestic and synanthropic species in the diet varies with cougar location along the wildland-urban gradient [28].

### Stage 1: Continuous-time Markov chain model

We used a Bayesian hierarchical CTMC model to evaluate drivers of cougar movement; this model is an extension of the model proposed by [19] and allows for inference on movement rates and directional bias, as opposed to use, in continuous time and discrete space. The initial step in the CTMC framework is to estimate a continuous movement path from the observed data points. We used the functional movement model developed by [13] to account for measurement error and predict locations every 10 minutes for the selected 2 weeks of each month. The functional movement model can be implemented using the fmove.bayes function in the ctmcmove package [41]. The smoothness of the imputed paths can be controlled using the arguments associated with the precision matrix and the shape and scale parameters of the inverse gamma prior for the partial sill parameter of the spline basis coefficients. The details of the functional movement model are beyond this manuscript, but for the purpose of reproducibility we note that we used prior knowledge to fix the standard deviation of the measurement error to $\sqrt{\log \left(10/4\right)}$, and modeled the variance of the basis functions using a prior consisting of a lag two conditional autoregressive precision matrix with the partial sill modeled as an inverse gamma with shape and scale parameters equal to one. Additional details of the functional movement model can be found in [13, 14, 41, 42].

Although the CTMC method is computationally efficient in terms of speed, there are trade-offs between the duration of the time-period of interest, the temporal resolution of the path interpolation, and the spatial resolution of the rasters, which together can create extremely large and difficult to store data frames. In addition, when the latent variable formulation is used, the discrete cell sequence must be contiguous, meaning that the spatial and temporal resolutions must match (e.g., an individual cannot move further than one grid cell between time points). In our analysis, the time-period of interest (2 weeks), temporal resolution (10 min intervals), and spatial resolution (100-m by 100-m or 1 ha), were selected to focus on fine-scale movement decisions. Additionally, crossing a 100 m^2^ area encompasses a cougar’s ability to move over a ten-minute interval [17]. To account for uncertainty in the true movement path, a random subset of imputed paths from the posterior predictive distribution of the movement model were spatially discretized to a latent variable formulation with a cell size of 100-m by 100-m, which was the lowest resolution among the available covariates.

The CTMC model consists of a product of two components: the time an individual spends in a grid cell and the direction that an individual moves when it leaves a grid cell. The time an individual spends in a grid cell (motility) is exponentially distributed, such that a large rate parameter corresponds to fast movement out of the cell. When an individual leaves a grid cell, the probability that they move to a particular neighboring grid cell (directionality) is the ratio between the movement rate into that cell and the sum of the movement rates into all neighboring grid cells. Therefore, higher proportional rates indicate directional bias in movement. Thus, movement rate parameters, which are a function of covariates (i.e., landscape variables that correspond to the position of the cell on the landscape), control both motility and directionality. Hanks et al [19] showed that the likelihood of the CTMC model (i.e., the product of the motility and directional components) for movement can be expressed as a Poisson GLM using a latent variable formulation.

In the latent variable formulation, each transition corresponds to four data points (the four neighboring grid cells); the response variable is equal to one if the neighboring grid cell is the cell that the individual transitioned into and zero otherwise. Modeling the latent variables (zeros and ones) as Poisson random variables with an offset for the amount of time an individual spends in a grid cell results in a likelihood that is equivalent to the CTMC likelihood. This allows inference to be obtained using standard GLM software, and the R package ctmcmove facilitates creation of the CTMC latent variable formulation [41]. Full CTMC details are available in Additional file 1.

Using multiple imputed paths accounts for the uncertainty in the true path of the individual and is a process version of multiple imputation [15, 18, 19, 43], a method frequently used for missing data [44]. Process imputation is more computationally efficient than using the entire posterior distribution, but still approximates the uncertainty associated with the unobserved path. We generated 30 imputations for each individual [19, 43], using 20 imputations to fit the models for individual-and population-level inference on transition rates and 10 imputations to calculate the posterior predictive score that was used to select regularization terms. Regularization shrinks the effect of unimportant covariates toward zero to prevent over-fitting and, in a Bayesian context, this is achieved by using an informative prior for the coefficients [45].

### Stage 2: Poisson models for movement inference

We used a hierarchical generalized linear model (H-GLM) for individual- and population-level inference on average cougar behavior, as measured by movement rates and directional bias, as a function of landscape features. Because cougars and humans are active at different times throughout the day, we proposed an additional model, a hierarchical generalized additive model (H-GAM), to account for individual- and population-level diel time-varying behavior. Covariates were centered and scaled to the individual, meaning that the coefficients are relative to the mean and standard deviation of the values that each individual encountered during a given two-week period. This is similar to the idea proposed by [2], where selection was determined by comparing some measure of usage and availability of a landscape feature on an individual basis. The hierarchical component of the model allows individual-level responses to vary around a population-level mean response, where both the individual and population-level estimates are obtained simultaneously.

In the CTMC framework, the response variables, *z*_*ij*_, were a sequence of zeros and ones, where *z*_*ij*_~Poisson(*λ*_*ij*_), for *i* = 1, …, *T* and *j* = 1, …, *J*, where *T* was the total number of cell transitions, and *J* was the number of individuals. Landscape covariates were incorporated using the log link function, such that $\log \left({\lambda}_{ij}\right)=\log \left({\tau}_{ij}\right)+{\mathbf{x}}_{ij}^{\prime }{\boldsymbol{\beta}}_j$. The residence times were represented by the constants *τ*_*ij*_, and the landscape variables by **x**_*ij*_. The parameter ***β***_*j*_ was a vector of *P* individual-level coefficients that arose from the population-level distribution ${\boldsymbol{\beta}}_j\sim \mathcal{N}\left({\boldsymbol{\mu}}_{\beta },{\boldsymbol{\Sigma}}_{\beta}\right)$. The covariance matrix, ${\boldsymbol{\Sigma}}_{\beta}\equiv {\sigma}_{\beta}^2\operatorname{diag}\left(\boldsymbol{\phi} \right)$, where the vector ***ϕ*** scaled the value ${\sigma}_{\beta}^2$ to each coefficient. The vector of scaling parameters consisted of a one for *p* = 1 (*ϕ*_1_ = 1) and was modeled as $\log \left({\phi}_p\right)\sim \mathcal{N}\left(0,0.04\right)$ for *p* = 2, …, *P*. The population-level distribution had a mean that was modeled with a multivariate normal distribution ${\boldsymbol{\mu}}_{\beta}\sim \mathcal{N}\left(\mathbf{0},{\sigma}_{\mu}^2\mathbf{I}\right)$, where **I** is the identity matrix. Both ${\sigma}_{\beta}^2$ and ${\sigma}_{\mu}^2$ were used as regularization terms, where ${\sigma}_{\beta}^2$ was selected a priori and ${\sigma}_{\mu}^2=0.1$, to shrink the coefficients toward zero; this prevented over-fitting and allows for correlated predictors [45].

The H-GAM was formulated as a varying coefficient model [46], where the response to covariates varied over space or time. By expanding the landscape covariates with a basis function [47], we created a new vector, **v**_*ij*_, that was the Kronecker product of the *P* length vector of covariates, ${\mathbf{x}}_{ij}^{\prime }$, and the *Q* length vector of the values of the basis at the time of transition *i*, **w**(*i*). For diel movement, we used cubic cyclic spline basis functions (**w**(*i*)), because they constrain the start and end points of the varying coefficients to be equal, which is an important property for time spans that are cyclic in nature. The GAM for hourly movement was similar to the GLM, except $\log \left({\lambda}_{ij}\right)=\log \left({\tau}_{ij}\right)+{\mathbf{v}}_{ij}^{\prime }{\boldsymbol{\alpha}}_j$, where ***α***_*j*_ was a vector of length *PQ*. Each parameter in ***α***_*j*_ was the collective effect of the basis function and the corresponding covariate at the time of transition *i*. Using the vector **w**(*i*), ***α***_*j*_ can be back-transformed to obtain the time-varying effect of the covariate. In the hierarchical framework ${\boldsymbol{\alpha}}_j\sim \mathcal{N}\left({\boldsymbol{\mu}}_{\alpha },{\boldsymbol{\Sigma}}_{\alpha}\right)$, where ${\boldsymbol{\Sigma}}_{\alpha}\equiv \operatorname{diag}\left({\sigma}_{\alpha}^2\boldsymbol{\phi} \right)$. The vector ***ϕ*** again reduced the number of parameters we need to select a priori by scaling the ${\sigma}_{\alpha}^2$ term to each parameter, and ${\boldsymbol{\mu}}_{\alpha}\sim \mathcal{N}\left(\mathbf{0},{\sigma}_{\mu}^2\mathbf{I}\right)$. Both ${\sigma}_{\alpha}^2$ and ${\sigma}_{\mu}^2$ served as regularization terms, where ${\sigma}_{\alpha}^2$ was selected a priori and ${\sigma}_{\mu}^2=0.1$.

Finally, to assess whether males and females exhibited different amounts of temporal variation in their response to potential movement drivers, we fit the GLM and GAM models to males and females separately for each time period. This resulted in four models: 1.) a GLM fit to all individuals, 2.) a GAM fit to all individuals, 3.) a GLM fit to females and a GAM fit to males, 4.) and a GAM fit to females and a GLM fit to males. We calculated the posterior predictive score for each model (i.e., the sum of the posterior predictive score for the models fit to males and females separately) and compared the scores across models within each month.

Models were fit using a Markov Chain Monte Carlo (MCMC) algorithm written in R [48]. We performed adaptive tuning over an initial 50,000 MCMC iterations. We used the selected tuning parameters as constants in the subsequent 50,000 iterations that were used to calculate the posterior predictive score for the a priori regularization parameter grid-search. The final models were fit using 100,000 MCMC iterations with a burn-in period of 10,000 iterations.

### Landscape covariates

Each covariate can be included as either a motility or directional driver of movement in the CTMC model. Motility covariates are based on the value of the grid cell that the individual is in currently and control the absolute rate of movement; positive coefficients indicate faster movement with increasing values of the covariate (and slower movement with decreasing values), and negative coefficients correspond to faster movements with decreasing values of the covariate (and slower movement with increasing values). Directional covariates account for the correlation between movement and the gradient of a covariate and contribute to the probability that an individual moves toward a grid cell. The directional drivers were calculated such that a positive coefficient indicates that individuals move predominantly in the direction that the covariate decreases (decreasing distance, such that they orient toward a feature), whereas a negative coefficient indicates that individuals move in the direction that the covariate increases (increasing distance, orient away from a feature). All rasters were aggregated to a 100-m by 100-m resolution, which is within the distance that a cougar might typically move over a ten-minute interval [17]; individuals cannot skip grid cells (enter a non-neighboring cell), therefore the spatial resolution of the rasters should reflect our prior knowledge about movement speeds.

We hypothesized that a number of landscape covariates may contribute to transition rates and directional bias of cougars: mule deer (*Odocoileus hemionus*) utilization (as a proxy for availability), distance to nearest potential kill site, distance to nearest structure, distance to nearest road, elevation, heat insolation load index, and topographic wetness. We also used an autoregressive parameter to account for directional persistence, or an individual’s tendency to move in the direction in which it was already moving [19].

Prey availability is a driving factor in cougar habitat selection. For example, cougars in western Washington used areas where suspected prey availability was high, such as low-elevation, early successional forests, and areas near water [21], and [27] observed cougars foraging in areas with high mule deer utilization. We approximated prey availability using two covariates: annual mule deer utilization and nearest potential kill site. The model averaged prediction for mule deer utilization [49] approximates prey availability given a suite of landscape covariates. We hypothesized that cougars would move slower in areas with high values for mule deer utilization and orient toward areas of high mule deer use during crepuscular and nocturnal movements [21, 27, 50]. Blake et al [51] found that many of the landscape variables that contribute to the location of predation events were the same as those contributing to non-predation habitat use, which led them to determine that cougars spend the majority of their time moving across the landscape in hunting mode. Including the location of a potential kill site may act as a proxy for unmeasured landscape variables and non-mule deer prey presence. In addition, potential kill sites represent known spatially recursive behavior based on memory and perception of the landscape [52–54]. Memory and recursively used locations have been incorporated into resource-selection analyses using individual-level intensity distributions [55, 56] and model-based Dirichlet processes [57]. Potential kill sites were determined using a clustering algorithm on the GPS points, where a location was classified as a potential kill site if two or more GPS locations, occurring between the average time of sunset and sunrise for each two-week period, were found within 200 m of the site within a six-day period (modified from [50, 58]). We calculated the distance (m) to nearest potential kill site identified within the two-week period to account for dependence in the movement process due to the known temporary activity centers induced by the potential kill sites. Up to nine potential kill sites were identified for each individual during the observation window (two-weeks). Although the data were used to generate the clusters, the CTMC model is not assessing resource use, but is determining whether speed and directionality vary as a function of the location of the clusters (e.g., we would not detect a response if they were not correlated with variation in movement). We expected individuals to move faster as distance to potential kill site increased, because decreasing distance may correspond to an individual returning to a cached kill, and caches are more often located in areas of high vegetation cover [59].

We calculated distance to nearest structure (m) as the Euclidean distance to the nearest man-made roofed structure [49]. Distance to road was calculated using major roads data (i.e., a major highway primarily for through traffic usually on a continuous route and streets whose primary purpose is to serve the internal traffic movement within an area) obtained from Colorado Department of Transportation. Due to increased human activity around structures and roads, we expected cougars to move faster when closer to roofed structure and distance to nearest road [17, 33, 60]. However, females may respond less to structures and roads than males, given that there may be additional factors, such as food limitation and offspring, which drive them to tolerate human-modified landscapes [37, 61]. We also expected there to be high temporal variability in the response to structures, because individuals have been observed avoiding areas of anthropogenic activity less at night, while avoiding contiguous forest habitat less during the day [22].

We used a digital elevation model (Fig. 1) to characterize elevation. Blecha et al [27] found that cougars avoided foraging in higher elevations, but [37] observed cougars selecting for higher elevations in developed areas. We expected cougars to show high temporal variability in their directional response to elevation, with cougars moving toward lower elevations when they are hunting (main prey is concentrated in lower elevations) and toward increasing elevations at other times. We used a raster based on the continuous heat insolation load index ([62], modified from [63]), to measure the accumulation of solar radiation at that location over the course of a year (MJ/cm^2^/yr). Heat insolation is higher on south-facing slopes that are more xeric and open than north-facing slopes [64]. Cougars have been observed using less rugged terrain for travel [17], selecting for south-facing slopes containing shrubs [22], and avoiding foraging on north-facing slopes [28]. Therefore, we expected that cougars may orient toward areas of high heat insolation, but move quickly through them. The topographic wetness plus metric (TWI+) predicts soil moisture based on slope, as originally described by [65], and aspect, as modified by [66]. Because cougars have been observed selecting for and hunting in riparian areas [21, 33, 60, 61], we expected cougars to move slowly in areas of high topographic wetness and demonstrate temporal variability in their directional response (toward areas of increasing topographic wetness when hunting).

We also analyzed a subset of individuals and the interaction between housing density and their response to deer utilization and distance to nearest kill site. Despite cougars demonstrating avoidance of high housing densities while foraging (locations preceding a successful kill and following previous prey handling), kill sites were positively related to housing density [27]. In addition, the temporal variability in the response to anthropogenic structures that was observed by [22] was stronger for cougars in rural, rather than wilderness, areas. Therefore, these are the two variables that we expected to vary most with housing density due to the potential trade-offs between increased prey abundance but increased mortality risk. To determine the effect of housing density on the response of cougars to deer utilization and potential kill sites, we discretized the landscape into developed (< 10 acres/unit) and undeveloped areas (Fig. 1). Only 13, 15, and 17 individuals for April, June, and October, respectively, were used in the secondary analyses because the remaining individuals did not spend time in developed areas in the selected two-week periods. We were unable to evaluate an interaction in the H-GAM (time-varying) framework due to high variability in the percentage of locations for each individual that were classified as occurring in undeveloped areas.

## Results

**Fig. 2:**
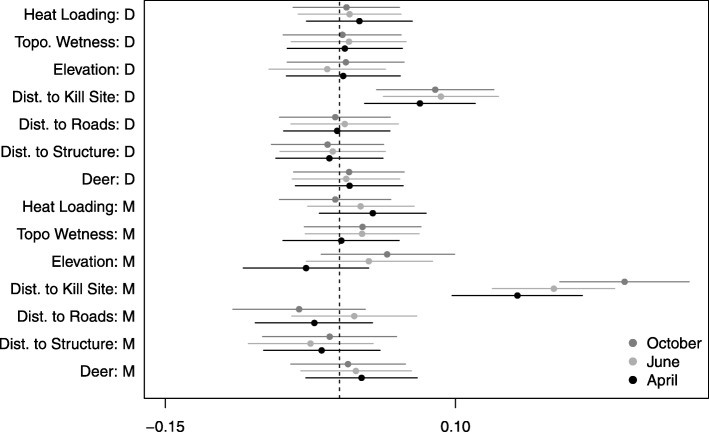
The mean and 95% credible intervals for the population-level mean effects of landscape covariates on movement rates (M) and directionality (D) of cougar movement in the Colorado Front Range for two-week periods in April, June, and October 2011

**Fig. 3:**
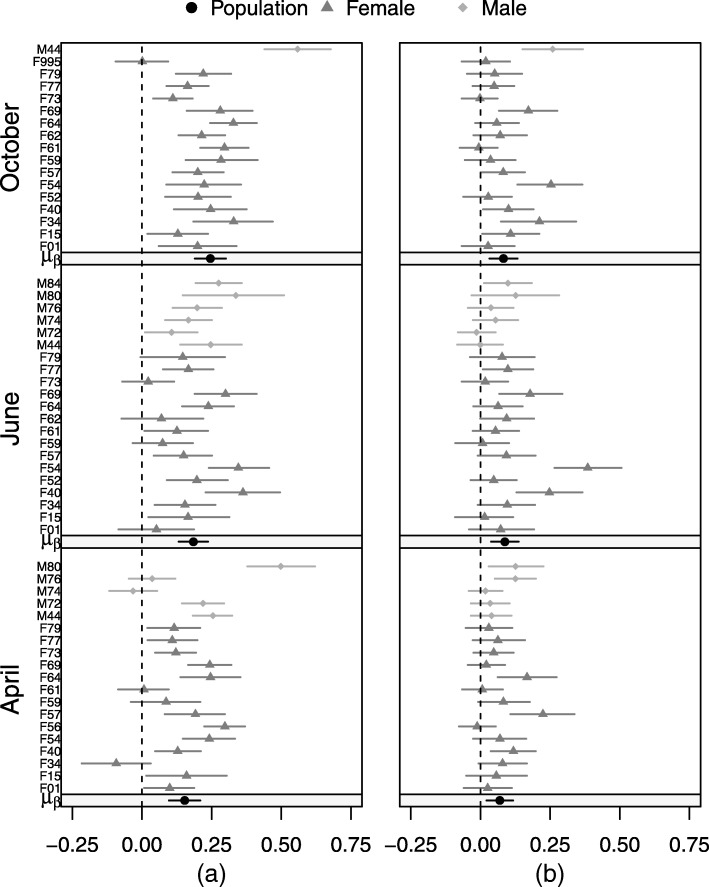
Posterior means and 95% credible intervals for the individual- and population-level static effects of distance to nearest potential kill site on motility (Fig. 3**a**) and directionality (Fig. 3**b**) of cougar movement in the Colorado Front Range for two-week periods in April, June, and October 2011

There was no detectable effect of many of the landscape covariates on average motility or directionality at a population-level (Fig. 2). However, distance to potential kill site emerged as the primary driver of both motility and directionality in the GLM framework (Figs. 2 and 3). As individuals increased their distance from a potential kill site, their transition rate increased (Fig. 3a). In addition, individuals oriented movement toward their potential kill site (Fig. 3b). We also detected significant directional persistence (95% CI for April: 1-1.15, June: 1.03-1.16, and October: 1.07-1.20), or residual autocorrelation, indicating that individuals tended to continue moving in the direction they had previously been moving, after accounting for landscape features.

**Fig. 4:**
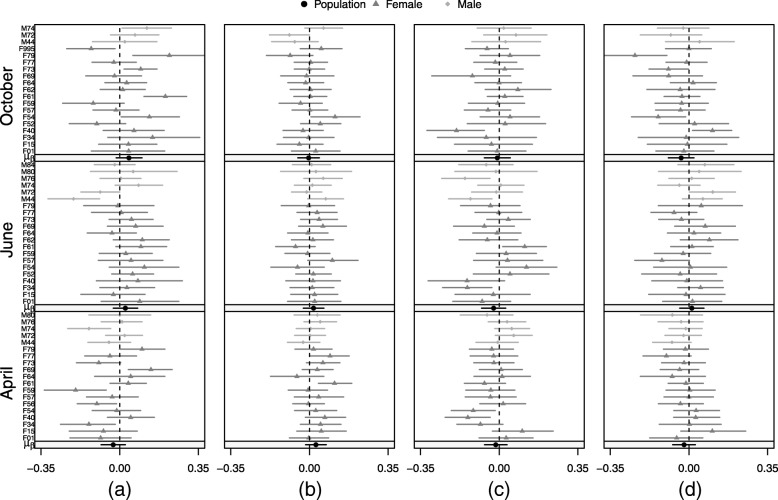
Posterior means and 95% credible intervals for the individual- and population-level static effects of elevation (Fig. 4**a**), heat loading (Fig. 4**b**), distance to nearest roofed structure (Fig. 4**c**), and distance to nearest road (Fig. 4**d**) on cougar motility in the Colorado Front Range for two-week periods in April, June, and October 2011

Of the remaining potential drivers of movement, the largest seasonal differences were observed in the effect of heat loading, elevation, distance to nearest roofed structure, and distance to nearest road; however, the 95% credible intervals consistently overlapped zero (Fig. 2). Based on the posterior mean, individuals were observed moving slower at higher elevations in April, but faster in June and October (Fig. 4a). In contrast, individuals moved faster than average in areas where heat loading was high in April, and to a lesser degree, June, but moved slower with higher heat loading in October (Fig. 4b). We detected a consistent seasonal effect of distance to structure, but the posterior mean was negative, meaning that individuals moved faster as distance to structure decreased (Fig. 4c). A similar pattern was detected with distance to roads, however the effect became positive in June, with individuals moving faster as distance to roads increased (Fig. 4d). In most cases, individual-level uncertainty tended to be high, with a few individuals showing statistically significant responses despite a non-significant population-level response (Figs. 3, 4).

**Fig. 5:**
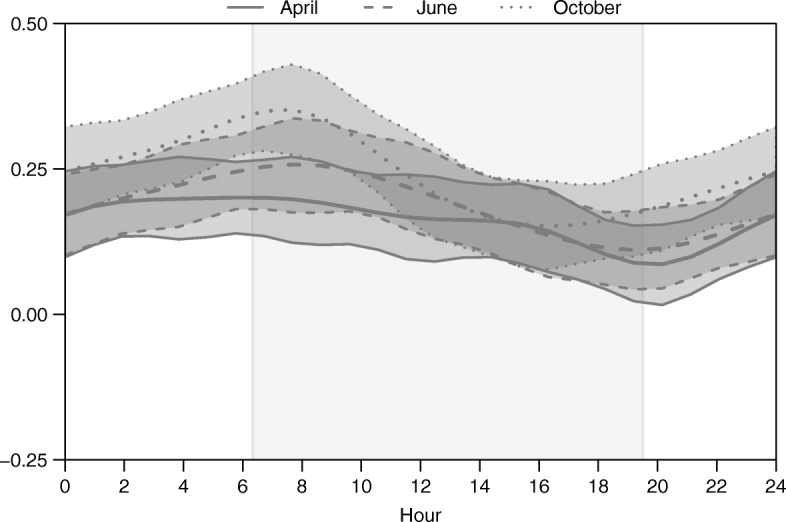
Posterior means and 95% credible intervals for the population-level diel time-varying effect of distance to nearest potential kill site on cougar motility in the Colorado Front Range for two-week periods in April, June, and October 2011. The gray box represents 0630 h to 1930 h.

**Fig. 6:**
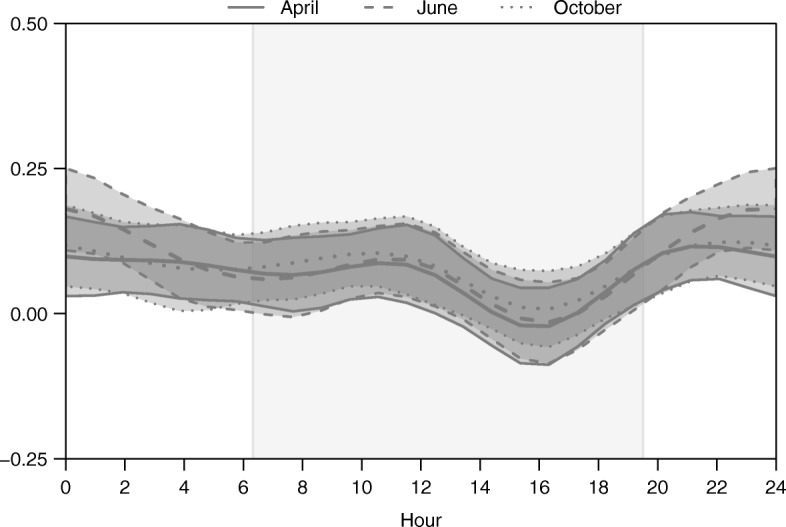
Posterior means and 95% credible intervals for the population-level diel time-varying effect of distance to nearest potential kill site on directionality of cougar movement in the Colorado Front Range for two-week periods in April, June, and October 2011. The gray box represents 0630 h to 1930 h

Our results suggest that distance to nearest potential kill site was also the predominant motility and directionality driver in the diel time-varying framework (H-GAM; Figs. 5 and 6). However, the strength of the motility response to distance to nearest potential kill site varied over time and with seasons. The strongest motility response occurred around dawn, decreased steadily during daylight hours, and then increased around dusk (Fig. 5). The magnitude of this variation was strongest in June and October, and weakest in April (Fig. 5). The strength of the directional bias toward potential kill sites also varied through time, but was consistent across seasons (Fig. 6). The evidence for an effect of potential kill sites on directionality suggests that individuals orient less toward their kill site during daylight hours, and may even orient away from their potential kill sites during late afternoon (Fig. 6).

**Fig. 7:**
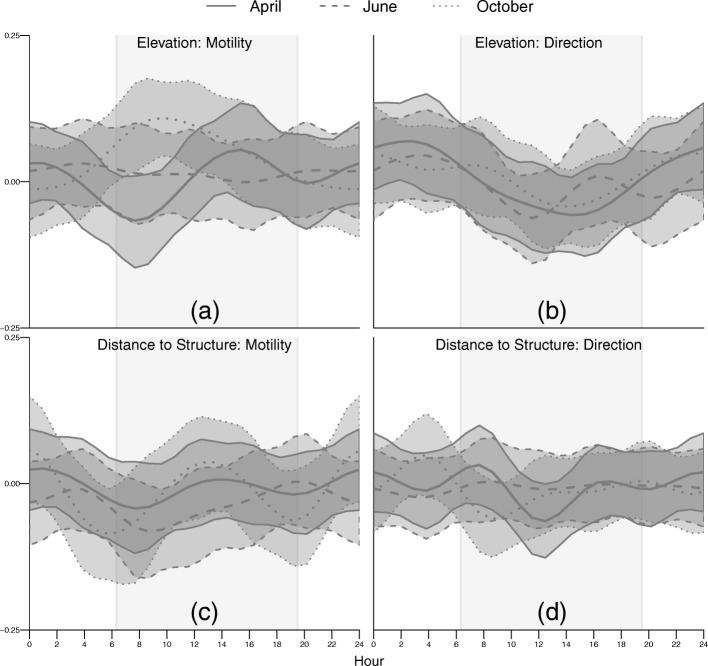
Posterior means and 95% credible intervals for the population-level diel time-varying effect of elevation (Fig. 7**a**, **b**) and distance to nearest roofed structure (Fig. 7**c**, **d**) on motility and directionality of cougar movement in the Colorado Front Range for two-week periods in April, June, and October 2011. The gray box represents 0630 h to 1930 h

While the 95% credible intervals overlapped zero for much of the day, we detected modest temporal responses in both motility and directionality to elevation and distance to nearest structure (Fig. 7). The motility response to elevation varied seasonally, as in the GLM framework. The average negative response to elevation observed in April (Fig. 4a) was reflected in a negative response to elevation around dawn (individuals move slower as elevation increases), with a slightly positive response later in the day (Fig. 7a). We observed little time variation in June, but the pattern observed in October was the opposite of April, with individuals moving faster with increasing elevation around dawn, with a decreasing effect through the rest of the day (Fig. 7a). Individuals moved toward higher elevations mid-day and toward lower elevations at other times, a pattern that was consistent across seasons (Fig. 7b). The strongest negative effect of distance to structure on motility (individuals move faster as distance decreases) occurred around dawn and dusk for all seasons (Fig. 7c). The effect on directionality was less consistent, with orientation toward roofed structures just after dawn, followed by orientation away from structures, in April and June; this pattern shifted toward pre-dawn in October (Fig. 7d).

**Fig. 8:**
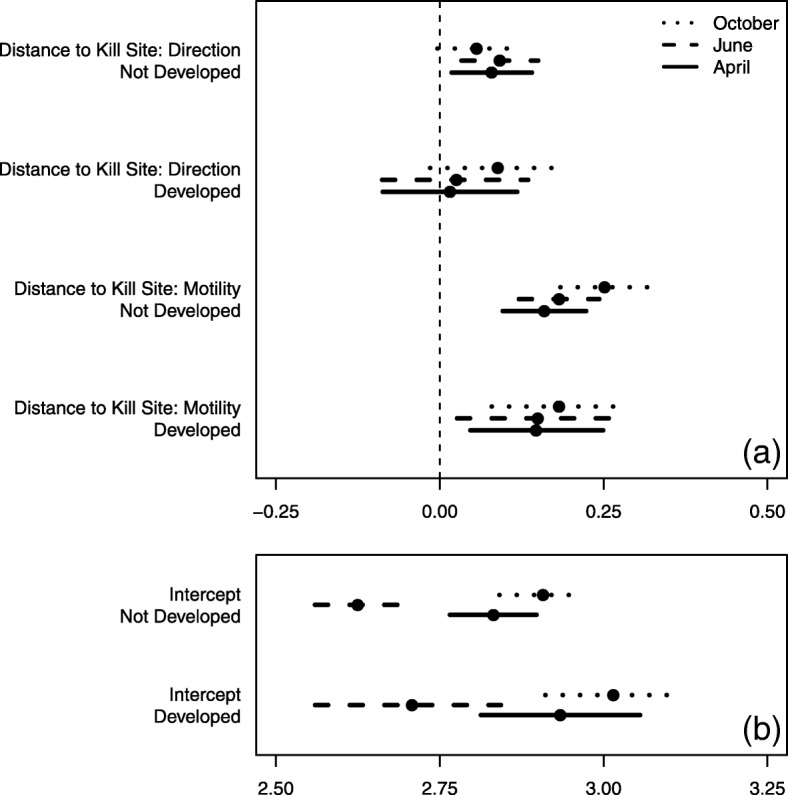
Posterior means and 95% credible intervals for the population-level effect of distance to nearest potential kill site on motility and directionality of cougar movement (Fig. 8**a**) and average movement rate (Fig. 8**b**) as a function of development (developed being < 10 acres/unit) in the Colorado Front Range for two-week periods in April, June, and October 2011

In addition, we did not see evidence for an interaction between development and deer utilization, which remained a statistically insignificant driver of cougar movement rates and directionality in both the H-GLM and H-GAM models. The positive effect of distance to potential kill site on speed (faster as distance to kill site increases) and directional bias (more orientation toward the kill site) was consistent between developed and undeveloped areas (Fig. 8a). However, we detected a difference in average movement rate between the two areas, with individuals in each month moving faster in developed areas (Fig. 8b).

**Fig. 9:**
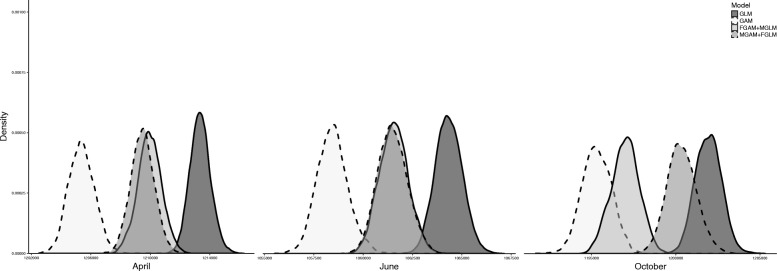
Posterior predictive score distributions for the model set, where smaller values indicate better predicting models. The GAM fit to all individuals performed the best in terms of predictive ability for all months

Finally, the H-GAM for both sexes was the best model in terms of predictive performance across all months, whereas the GLM performed the worst (Fig. 9). The models that were a mixture of a GAM and GLM, varying by sex, were generally equivalent (Fig. 9). The largest difference between the two sex-varying models was observed in October, when the better of the two models included time variation for males and no time variation for females (Fig. 9).

## Discussion

### Fine-scale cougar movement

The observed response to distance to nearest potential kill site over a short time period is potentially due to cougars returning to the carcasses (e.g., spatial memory; [52–54]) and unmeasured fine-scale covariates related to landscape features that increase the likelihood of a successful hunting attempt. Blecha et al [27] found that kill sites, compared to preceding locations, occurred more frequently in areas with higher housing densities and lower topographic positions, such as drainage areas, despite drainage areas having lower prey availability. We did not measure hunting success, but we did find that cougars moved toward lower elevations at dusk, when cougars are likely to hunt or return to a carcass. We found that individual response to nearest potential kill site was variable within the two-week period and across individuals; this is likely a function of timing of successful kills and the size of the prey item, with stronger positive responses being correlated with larger prey (as individuals return to the site over a longer period of time). In addition, the variation in motility across months may also be a function of the available prey items during a given season. We observed the weakest response to kill sites during April, before mule deer fawns are born [38]. We also observed an increasingly strong positive response to distance to kill site from dusk to dawn, implying that, from dusk to dawn, individuals moved increasingly faster the farther away they were from a potential kill site.

Our results indicated that cougars moved slightly faster in areas with a higher heat insolation load index in April compared to June and October. These areas correspond to xeric, south-facing slopes, which, in the montane zone of the Front Range, mostly consist of open stands of ponderosa pine, compared to the more dense north-facing slopes [64]. The more open forest floor may facilitate cougars using south-facing slopes as travel corridors, leading to greater transition rates. Similarly, [17] found that cougars used less rugged terrain than the surrounding area while traveling, while [22] found that cougars selected for south-facing slopes and areas with shrub habitat. The monthly difference in effect size for the response to heat loading may be related to seasonal changes in vegetation (shrub cover may be denser in June, reducing speed) or a product of unobserved weather patterns (e.g., more snow on north-facing slopes could lead to a greater tendency to use south-facing slopes as corridors). Similarly, we also observed slower movements at high elevations in April compared to June and October; high elevation areas of the Front Range may still contain snow in April, which could result in slower movement rates at high elevations. The time-varying directional response to elevation indicates that individuals are moving to higher elevations during the day, and then toward lower elevations at night. Blecha et al [27] found that predation events, which typically occur at night, occurred at lower elevations, which may explain the temporal pattern we observed. The difference among seasons for the dawn motility response (slower [faster] at high [low] elevations in April, faster [slower] at high [low] elevations in October) might also be a response to unobserved fine-scale vegetation changes or dietary shifts.

The documented response of cougars to disturbed and developed landscapes varies in the literature, and is likely a function of the level of disturbance encountered and how disturbance was quantified. For example, [21] found no difference in cougar movement rates in wildland and residential areas throughout the day. However, [22] observed that cougars avoided developed landscapes, while also noting a temporal shift in usage of those areas, with cougars avoiding areas near development more during the day. On average, we observed a more negative relationship to distance from structures at dawn and dusk compared to mid-day and evening (i.e., individuals moved faster when closer to structures during dawn and dusk than mid-day and evening, when there was a slight positive relationship between speed and distance to structure), which could be explained by increased human activity caused by the start and end of the workday. However, the uncertainty was fairly large for the diel effect of distance to nearest roofed structure, despite subtle positive and negative shifts. These minor differences, and an overall lack of consistent response, could be explained by unmeasured spatial and temporal relationships, such as individual interactions, fine-scale human disturbance (e.g., recreational activities, noise, and construction), and individual risk-avoidance strategies [67]. For example, [37] found that cougars showed stronger avoidance of more consistent sources of anthropogenic disruption, such as neighborhoods, than intermittent sources, such as low-traffic roads. We detected a faster average movement rate in areas with more development, which is consistent with work by [17] and [68], who found that individuals expended more calories and moved further in developed areas, and [69] also found that large mammal movement rates vary as a function of local conditions. Distance to roads and structures may not be adequate proxies for how cougars perceive anthropogenic disturbance, and alternative measures (e.g. density or categorical variables), or responses (actual road crossings, e.g., [70]), may be more relevant for cougar movement behavior.

Other studies have detected significant individual variation [21, 37], and [61] and [37] found that selection differed between males and females. We did not see consistent sex-specific responses to covariates, which could be due to the timing of the observations; for example, females may respond differently to males when breeding, but similarly at other times. In addition, males were underrepresented in our sample. Some of the unexplained individual variation could be due to the amount of anthropogenic landscape features each individual was likely to encounter in their movements [22, 61], as opposed to the amount of development in the immediate vicinity during a given movement. Benson et al [61] also hypothesized that the amount of development in many studies of cougar habitat selection has been too low to cause cougar behavioral changes.

We propose that our findings regarding a lack of evidence for significant landscape drivers of movement may have three potential biological causes. First, cougars are generalists, therefore, they are expected to demonstrate less habitat selection at the landscape scale than a habitat specialist would [71]. Though we were assessing movement, generalists may likewise demonstrate less variation in speed and directionality as a function of the landscape than would a specialist, or the risks and rewards present in the Front Range are not significant enough to cause a detectable response in behavior. In addition, individuals moving within an established home range, such as in this study, may be acclimated (demonstrating minimal change in behavior) to the disturbances that they encounter during daily movements. Second, significant individual-variation within a 24-h period would make determining a consistent population-level response difficult. Individual variation can occur across and within individuals, and may be a function of the unmeasured internal state of the animal (breeding status, body condition, and energetics), or external factors (interactions with other individuals, fine-scale landscape features, and unmeasured brief disturbances). Finally, cougar movement may correspond to, or interact with, lower frequency environmental variation (e.g., weather patterns and food availability). Comparing behavior among years could be used to assess seasonal consistency in observed patterns; however, due to the difficulty of performing multiple comparisons of time-varying effects, a study of among year differences would likely need to focus on a particular season of interest.

### Modelling framework

Many of the hypothesized movement drivers did not have a consistent statistically significant relationship with movement, while other studies on cougars in the same geographic area have found strong effects for landscape variables on cougar resource use while hunting [27]. However, unlike RSFs and traditional SSFs (not integrated, e.g. [10]), the CTMC framework is measuring the effect of landscape variables on speed and directionality, not habitat selection. For example, cougars may select for areas with high mule deer use [27], but cougars may not alter their speed based on the amount of mule deer usage. Although there has been a recent development that uses the limiting distribution of a CTMC to estimate utilization density (home-range; [20]), the relationship between changes in speed and directionality and habitat selection may vary by species. In addition, although multiple imputation appropriately accounts for the uncertainty in the unobserved true path, it does introduce an additional source of variation that is not accounted for when using only the observed locations. In addition, the variation among imputed paths for each individual will increase with measurement error, because there is less information to constrain the realizations of the true, unobserved path. Although measurement error was minimal in our study, the added uncertainty introduced by the multiple imputation framework may make it difficult to detect statistically significant effects.

The varying coefficient modeling framework, implemented in this study as a GAM, can reveal hidden process dynamics [72] and allows for complex nonlinear patterns that would be difficult to model in a traditional framework (e.g., [73]). While we expanded each parameter in to the temporal space, one could make each covariate a function of another parameter, such as a different temporal predictor (e.g., time since kill) or another parameter in the model (e.g., distance to structure). Allowing the coefficients to vary in time (or another covariate space) can also improve the predictive ability of the model, as it did in our study. GLMs can mask time-varying responses to covariates (e.g., [74]), because the response variable is aggregated over the time period of interest. Therefore, if the response of an individual switches between positive and negative (faster or slower movement rates), the estimated response will be approximately zero. Studies have found that cougars use a broader range of habitats for nocturnal movements than for daybed locations [17] and demonstrate temporal variability in their response to anthropogenic landscape features [22]. Therefore, restricting analysis of locations to a particular temporal subset (e.g. day vs night) may not be indicative of all behavior [75]. The time-varying CTMC framework represents an important step forward in detecting latent temporal patterns in animal movement and is especially useful when behavior is known to vary in time.

## Conclusions

Recursive events, as measured by potential kill site locations, were identified as the primary driver of motility and directionality for cougars in the Front Range of Colorado. Observed cougars also moved faster, on average, in developed areas compared to undeveloped areas. Many other landscape features, including proxies for anthropogenic development, did not have a strong population-level effect on cougar movement, potentially due to unexplained individual-level variation. We did not detect a link between cougars that had reports of human conflict and response to development. Cougars have demonstrated different second- and third-order selection to roads in previous studies [33], therefore, nuisance individuals may select for, or end up in, home ranges near human development, but do not respond differentially to areas closer to development within their home range [76]. The high degree of individual variation suggests that, if agencies want to minimize human-wildlife conflict, a “one size fits all” approach to cougar management and conflict abatement will likely be unsuccessful and management options should be varied and flexible. A potential proactive mitigation of cougar conflict is to identify potential kill sites and orient recreation (e.g., trails, camp sites) away from those areas. Kill site identification tools, such as those developed by [27], could prove useful in this regard.

We observed temporal variation in the population-level response to some landscape features (potential kill site, elevation, distance to structure), which highlights the importance of considering time-varying effects of covariates on movement behavior. Time-varying effects may be particularly important to consider when animal behavior is known to vary in time and when temporally static covariates may contain uncharacterized temporal variation (e.g., roads that vary in traffic load according to time of day and season). This study is also the first hierarchical application of the CTMC modeling framework, and demonstrates its ability to provide computationally efficient inference on individual- and population-level drivers of animal movement behavior.

## Additional file

Additional file 1: Continuous-Time Markov Chain Model Details. Provides additional details on the CTMC model specification. (PDF 133 kb)

## Abbreviations

CPW: Colorado Parks and Wildlife
CTMC: Continuous-time Markov chain
H-GAM: Hierarchical generalized additive model H-GLM Hierarchical generalized linear model GPS Global positioning system
RSF: Resource selection function
SSF: Step selection function
