# Supplementary material for: Time-varying predatory behavior is primary predictor of fine-scale movement of wildland-urban cougars
Source: Mov Ecol. 2018 Nov 2;6:22. doi: 10.1186/s40462-018-0140-6 (PMC6214169; doi:10.1186/s40462-018-0140-6)
Supplement: Supplementary file 1 — Continuous-Time Markov Chain Model Details. Provides additional details on the CTMC model specification. (PDF 133 kb) [file 40462_2018_140_MOESM1_ESM.pdf]

Appendix A

Continuous-Time Markov Chain Model Details

We spatially discretize a posterior predictive continuous path from the movement model to the resolution of the rasters of interest and decompose it into two elements: $\mathbf{c}$, a state sequence consisting of the sequential grid cells (of N possible grid-cells) visited by the individual, and $\boldsymbol{\tau}$, a vector of residence times that describe how long the individual spent in each grid cell. It is important to note that individuals cannot skip grid cells (enter a non-neighboring cell), therefore the spatial resolution of the rasters should mirror our prior knowledge about movement speeds. We describe the cell sequence in terms of the transition rates $\boldsymbol{\alpha}$ where α_{i_j} is a parameter controlling movement from cell i to cell j that can be a function of spatial covariates:

$$\alpha_{ij} = e^{\mathbf{x}'_{ij}\boldsymbol{\beta}} \tag{1}$$

If we designate t as the tth observation in the state-sequence ($t \in T$), then the residence time τ_t is exponentially distributed with a rate equal to the sum of all α_{ij} (the total transition rate):

$$[\tau_t|\boldsymbol{\beta}] = \left(\sum_{j=1}^{N} \alpha_{ij}\right) e^{-\tau_k \sum_{j=1}^{N} \alpha_{ij}}. \tag{2}$$

In the above notation, $[\tau_t|\boldsymbol{\beta}]$ represents the probability distribution of the random variable τ_t given the parameters $\boldsymbol{\beta}$; this notation will appear again. We assume that it is impossible to move directly to non-neighboring cells, and therefore $\alpha_{ij} = 0$ for all j except for the cells adjacent to cell i.

When an individual transitions to a neighboring cell, the probability of transitioning to cell $c_{t+1} = l$ is

$$[c_{t+1} = l|c_t = i] = \frac{\alpha_{il}}{\sum_{j=1}^{N} \alpha_{ij}}. \tag{3}$$

Assuming independence, the joint likelihood is the product of the transition probabilities

and the residence times in the state sequence $\mathbf{c}$ is:

$$[\tau_t, c_{t+1} = l | c_t = i, \boldsymbol{\beta}] = \frac{\alpha_{il}}{\sum_{j=1}^{N} \alpha_{ij}} \left(\sum_{j=1}^{N} \alpha_{ij} \right) e^{-\tau_t \sum_{j=1}^{N} \alpha_{ij}} \tag{4}$$

$$= \alpha_{il} e^{-\tau_t \sum_{j=1}^{N} \alpha_{ij}} \tag{5}$$

Using a latent variable representation, where

$$z_{ij} = \begin{cases} 1, \text{if } j = c_{t+1} \\ 0, \text{if } j \neq c_{t+1} \end{cases} \tag{6}$$

and

$$[z_{ij}, \tau_t | \boldsymbol{\beta}] \propto \alpha_{ij}^{z_{ij}} e^{-\tau_t \alpha_{ij}}, \tag{7}$$

then the product of $[z_{c_t k}, \tau_t | \boldsymbol{\beta}]$ over all N is proportional to the likelihood of the observed transition:

$$[z_{ij}, \tau_t | \boldsymbol{\beta}] \propto \sum_{t=1}^{T} \sum_{j=1}^{N} \alpha_{ij}^{z_{ij} e^{-\tau_t \alpha_{ij}}}. \tag{8}$$

Additional details can be found in Hanks et al. 2015. The above process is parameterized with a single realization from the movement model. To avoid computational storage limitations, we use multiple imputation to account for the uncertainty in the path and make approximate posterior predictive inference on transition rates.

Literature Cited

Hanks, E.M., M.B. Hooten, and M.W. Alldredge. 2015. Continuous-time discrete-space models for animal movement. Annals of Applied Statistics 9:145165.
